# Brucine: A Review of Phytochemistry, Pharmacology, and Toxicology

**DOI:** 10.3389/fphar.2020.00377

**Published:** 2020-04-03

**Authors:** Lu Lu, Rui Huang, Ye Wu, Jin-Mei Jin, Hong-Zhuan Chen, Li-Jun Zhang, Xin Luan

**Affiliations:** ^1^Institute of Interdisciplinary Integrative Biomedical Research, Shuguang Hospital, Shanghai University of Traditional Chinese Medicine, Shanghai, China; ^2^Department of Pharmacology and Chemical Biology, Shanghai Jiao Tong University School of Medicine, Shanghai, China

**Keywords:** brucine, *Strychnos nux-vomica* L., phytochemistry, pharmacology effects, toxicity

## Abstract

Brucine, a weak alkaline indole alkaloid, is one of the main bioactive and toxic constituents of *Nux-vomica*. Modern pharmacology studies and clinical practice demonstrate that brucine possesses wide pharmacological activities, such as anti-tumor, anti-inflammatory, analgesic, and the effects on cardiovascular system and nervous system, etc. However, its central nervous system toxicity severely limits its clinical application. Herein, the physicochemical properties, pharmacological activities, and toxicity of brucine were reviewed, and the novel strategies to address the toxicity issues were discussed, aiming to bring new insights into further research and application of this active component.

## Introduction

Brucine is extracted from the seeds of *Strychnos nux-vomica* L. (Loganiaceae), which are commonly known as *Nux-vomica* (Maqianzi) with bitter taste and high toxicity. There are 190 species of *Strychnos nux-vomica* L. in the world, and mainly distributed in tropical and subtropical areas (Shi et al., 2017). *Nux-vomica* is widely used as medicine in many southern Asian countries. In China, *Nux-vomica* has a long history to use for the treatment of different kinds of ailments, such as dyspepsia, nervous system diseases, and chronic rheumatism (Guo et al., 2018).

Brucine and their nitrogen are the main constituents of *Nux-vomica*. Brucine is usually used as an anti-inflammatory and analgesic drug to relieve arthritis and traumatic pain. Recent years, brucine displayed excellent anti-tumor effect on various tumors (Li et al., 2018; Qin et al., 2018). For hepatocellular carcinoma, brucine could inhibit the proliferation of HepG2 cells through regulating calcium concentration and depolarization of mitochondria (Shu et al., 2013). In bone metastasis nude mice model of breast cancer, brucine might inhibit tumor angiogenesis, growth, and bone metastasis by down-regulating vascular endothelial growth factor (VEGF) expression (Li et al., 2012). And brucine could inhibit the growth and migration of colorectal cancer cells LoVo by regulating Wnt/β-catenin signaling pathway (Shi et al., 2018).

Although brucine has an impressive profile in pharmacology research, severe central nervous system toxicity is the main obstacle to its clinical application (Guo et al., 2018). The therapeutic window is quite narrow, and the reported lethal dose 50% (LD_50_) value of brucine was 50.10 mg/kg (Chen et al., 2013), which severely limits its clinical application (Chen et al., 2012). In order to better understand the pharmacological and toxicological effects of brucine and provide theoretical basis for its future application, we summarized the physicochemical properties, pharmacological activities, and toxicity of brucine here. Furthermore, the novel strategies to address the toxicity issues of brucine were also discussed.

## Physicochemical Properties, Extraction, and Purification of Brucine

### Physicochemical Properties

Brucine (2, 3-dimethoxystrychnidin-10-one,C_23_H_26_N_2_O_4_), a weak alkaline indole alkaloid, is white crystalline powder with a molecular weight of 394. It can be easily dissolved in organic solvents such as ether, chloroform, ethanol, and methanol but not in water.

**Figure 1:**
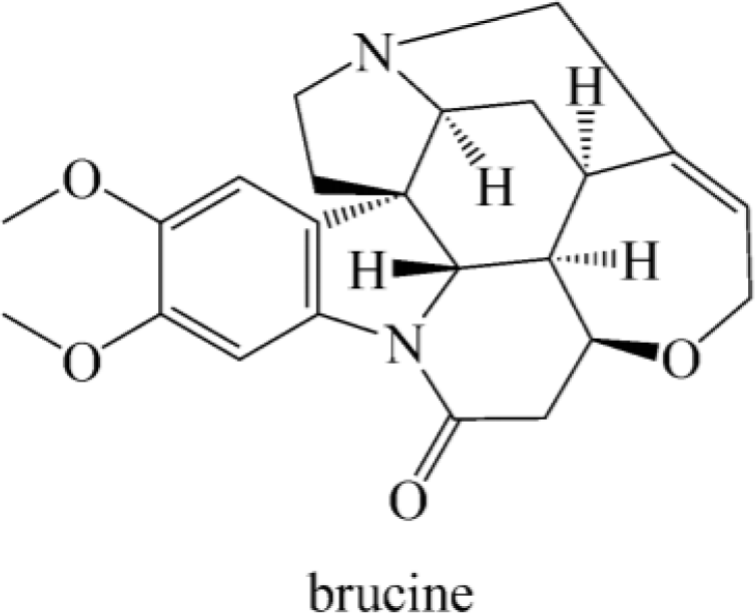
The structure of brucine.

Pelletier and Caventou first isolated brucine from the *Nux-vomica* in 1819 (Tang et al., 2009). Brucine is structurally related to strychnine so that could be used as a tool for stereospecific chemical syntheses and had been used as an enantioselective recognition agent in chiral resolution. In 1919, Groth reported that tetrahydrate was the solid form of brucine. The Cambridge Structural Database (CSD) has collected different crystal forms of brucine including two anhydrous forms (**Figure 1**) (Groom et al., 2016).

### Extraction and Purification

Some reports showed that brucine could be extracted from *Nux-vomica* powder by reflux extraction with acid water, ethanol, alkaline chloroform, or other solvents. Generally, extraction with ethanol has much better efficiency. When the total alkaloids of *Nux-vomica* processed by sand blanching were extracted by ammonia chloroform, water, acid water, or acid ethanol, the best effect was obtained through refluxing and extracting with 6-fold 50% alcohol (pH=5) for three times, 1 h for each time (Li et al., 2004). Considering acid ethanol would corrode equipment in industrial production which had low feasibility, this group then used neutral ethanol to extract raw brucine power. Meanwhile, microwave-assisted extraction method can improve the extraction rate of brucine. Although structure of brucine is similar to strychnine, brucine could be separated or purified by pH zone countercurrent chromatography or silica gel column chromatography (Li et al., 2010). Additionally, using silica gel column chromatography combined with semi-preparative high performance liquid chromatography (HPLC) to separate and purifying brucine was more simple and convenient (Wu et al., 2016a).

## Quantitative Analysis of Brucine

With the deepening understanding of the role of brucine, its application has been more and more extensive. As an effective and toxic component, brucine quantitative analysis is particularly important.

The content of brucine is usually determined by HPLC, but if the treatment methods of brucine are different, the results will be different. The content of brucine in different processed products and different origins of *Nux-vomica* was monitored by HPLC and the results showed that the content of brucine in processed products of *Nux-vomica* ranked from high to low were fried products, sand blanching products, vinegar boiled products, camphor urine soaked products, vinegar boiled sand fried products, vinegar soaked products, vinegar soaked sand fried products. On the other hand, according to the different habitats, the content of brucine ranged from high to low which were Vietnam, Guangxi, India, Yunnan, Myanmar, Sichuan, Hubei, and Hainan (Wang and Qi, 2017). Using reverse phase high performance liquid chromatography (RP-HPLC) to determine the content of brucine in GuizhiStrychni and Strychnine tablets is accurate, sensitive, and reproducible (Lou, 2016). Meanwhile, the contents of brucine in rat plasma and tissue could be determined by liquid chromatography tandem mass spectrometry (LC-MS/MS) (Qin et al., 2012b; Li et al., 2019).

## Pharmacological Activities

### Anti-Tumor Effects

Recently, a large number of studies have proved that brucine can significantly inhibit several tumor cells through different mechanisms. (**Table 1**) (Agrawal et al., 2011; Guo et al., 2018; Qin et al., 2018; Ren et al., 2019).

**Table 1:** The anti-tumor mechanisms of brucine.

Type of action	Model/Targeted cell	Mechanism	References
Apoptosis	Human monocytic leukemia cells THP-1, human chronic myeloid leukemia cells KCL-22, CRC cells Lovo	Regulate Bax/Bcl-2 balance and activate endogenous mitochondrial pathway	(Zheng et al., 2013; Xin et al., 2014; Han et al., 2016)
	Breast cancer cells MCF-7	Induce cells death in G2 phase and inhibit the expression of NF-κB subunit (p65)	(Serasanambati et al., 2015)
	Lung cancer cells A549, glioma cells U251, HCC cells HepG2	Inhibit the activity of NF-κB and COX-2 gene, reduce the expression of Bcl-2 and COX-2, increase the expression of Bax	(Deng et al., 2006; Zhu et al., 2012; Wang et al., 2015)
	Bone metastasis model of breast cancer in nude mice, MDA-MB-231 and MC3T3-E1 coculture	Regulate bone metastasis-associated factors such as MMP-2, CXCR4, RANKL, OPG	(Sun et al., 2017; Xu et al., 2019)
Metastasis inhibition	Breast cancer cells MDA-MB-231 and Hs578-T	Reverse EMT and Inhibit MMP-2/MMP-9	(Li et al., 2018)
	HCC cells	Inhibit the HIF-1 pathway	(Shu et al., 2013)
Intervention in cell signal transduction	CRC cells LoVo	Regulate the Wnt/β-catenin signaling pathway, inhibit angiogenesis by mediating the KDR signal pathway	(Luo et al., 2013; Shi et al., 2018; Ren et al., 2019)
	Multiple myeloma cells U266	Inhibit JAK-STAT signaling pathway	(Ma et al., 2013)

CRC, colorectal cancer; HCC, hepatocellular carcinoma; Bcl-2, B cell lymphoma/lewkmia-2; Bax, Bcl-2 associated protein; NF-κB, nuclear transcription factor-κB; COX-2, cyclooxygenase 2; CXCR4, chemokine (C-X-C motif) receptor 4; RANKL, receptor of NF-κB ligand; OPG, osteoprotegerin; EMT, epithelial-to-mesenchymal transition; MMP-2, matrix metalloproteinase-2; MMP-9, matrix metalloproteinase-9; HIF-1, hypoxia inducible factor-1; KDR, kinase insert domain receptor; JAK, janus kinase; STAT, signal transducer and activator of transcription.

#### Breast Cancer

Brucine could inhibit the bone metastasis of breast cancer by regulating the expression of bone metastasis-related factors such as matrix metallopeptidase 2 (MMP-2), chemokine (C-X-C motif) receptor (CXCR4), receptor of NF-κB ligand (RANKL), and osteoclastogenesis inhibitory factor (OPG) (Sun et al., 2017). Xu et al. also found that in the co-culture system of triple negative breast cancer cells MDA-MB-231 and murine osteoblasts MC3T3-E1, brucine could indirectly regulate osteoclasts by regulating the expression and secretion of OPG and RANKL, thus inhibiting osteoclast differentiation and bone absorption (Xu et al., 2019). Therefore, brucine can inhibit bone metastasis of MDA-MB-231 by regulating OPG/RANKL/RANK signal pathway.

By observing the effect of brucine on MDA-MB-231 and Hs578-T cell lines, as well as the expression of epithelial-to-mesenchymal transition (EMT) markers and matrix metalloproteinase (MMPs), studies found that the ability of migration, invasion or adhesion of MDA-MB-231, and Hs578-T cells decreased in a dose-dependent manner when treated with brucine (Li et al., 2018). These results proved the inhibitory effects of brucine on the bone metastasis of breast cancer. Moreover, brucine also could induce MCF-7 death in G2 phase and inhibit the expression of NF-κB subunit (p65) when used alone or in combination with gemcitabine (Serasanambati et al., 2015).

#### Liver Cancer

At present, brucine has been extensively studied in the treatment of hepatocellular carcinoma. Brucine inhibited the proliferation of HepG2 cells *in vitro* in a time- and dose-dependent manner. The mechanism might be that brucine first activated MAP kinase kinase-7 (MKK7) gene, and then the MKK7 kinase activated the pathway mediated by c-Jun N-terminal kinase (JNK) gene to induce apoptosis (Liang et al., 2017). Brucine continuously down regulated the expression level of HIF-1 response gene *in vivo* (Shu et al., 2013). Brucine could also inhibit the proliferation of HepG2 cells by inducing cell contraction, vesicle formation, and apoptotic body formation. At the same time, brucine significantly reduced the expression of cyclooxygenase-2 (COX-2) in HepG2 cells, but increased the expression of Caspase-3 and the activity of Caspase-3-like protease (Deng et al., 2006).

#### Hematological Tumor

Brucine also has certain inhibitory effect on hematological tumors. Xin et al. showed that brucine could inhibit the human monocytic leukemia cell line THP-1 cell growth in concentration- and time-dependent manners at the range of 50 to 400 ug/ml. Meanwhile, the expression of B cell lymphoma/lewkmia-2 (Bcl-2) gene was decreased while the expression of Bcl-2 associated protein (Bax) gene increased (Xin et al., 2014). Through regulating Bax/Bcl-2 balance and activating endogenous mitochondrial pathway,brucine couldalso induce the apoptosis of human chronic myeloid leukemia cell line KCL-22 in the concentration range of 50-400 ug/ml (Han et al., 2016).

#### Colorectal Cancer

It was showed that brucine was involved in the regulation of Wnt/β-catenin signaling pathway to inhibit the growth and migration of colorectal cancer cells LoVo *in vitro* and *in vivo* (Shi et al., 2018). Moreover, the inhibition of brucine on colon cancer proliferation was related to Wnt/β-catenin signaling pathway, in which the expression of dickkopf-related protein 1 (DKK1) increased significantly, while the expression of β-catenin decreased (Ren et al., 2019). Brucine could also inhibited the secretion of VEGF and the expression of mammalian target of rapamycin (mTOR) of Lovo cells, down-regulated the mRNA and phosphorylation protein expression of kinase insert domain receptor (KDR), protein kinase C α (PKCα), phospholipase C-γ (PLCγ), and v-raf-1 murine leukemia viral oncogene homolog 1 (RAF1), suggesting that brucine has the effect of inhibiting the angiogenesis by mediating the KDR signal pathway (Luo et al., 2013).

Brucine might inhibit the activation of signal transducer and activator of transcription (STAT3) phosphorylation in IL-6/STAT3 pathway to exert an antitumor effect on SW480 cells *in vitro* (Li et al., 2016). Meanwhile, brucine could induce apoptosis of Lovo cells in a dose-dependent manner by up-regulating Bax and Bcl-2, but down-regulating extracellular signal-regulated kinase 1/2 (ERK1/2), p38 and Akt protein phosphorylation (Zheng et al., 2013).

#### Other Cancer

In addition, brucine could significantly inhibit the proliferation of lung cancer cellA549 and induce apoptosis in a time-dependent manner by inhibiting the expression of COX-2 and releasing prostaglandin E2(PGE2) (Zhu et al., 2012). Brucine could also inhibit the proliferation of human lung cancer cell line PC-9 mainly by blocking the cell cycle at G0/G1 *via* down-regulating the expression of Cyclin D1, Cyclin E (Li et al., 2014).

When concentration of brucine was no more than 0.4 mg/ml, it might induce apoptosis of myeloma cells U266, and the effect of brucine on apoptosis was dose-dependent and time-dependent. RT-PCR was used to detect the changes of c-Jun expression after treatment with brucine or brucine combined with JNK specific inhibitor SP600125. It was found that brucine induced apoptosis of U266 cells through c-Jun phosphorylation. Therefore, brucine can induce apoptosis of U266 through JNK signaling pathway and c-Jun phosphorylation (Ma et al., 2013). Furthermore, experiments showed that brucine could reduce the expression of Bcl-2 and COX-2 in U251 glioma cells, up-regulate the expression of Bax, reduce the survival rate of glioma cells, and inhibit the growth of the xenografts *in vivo* (Wang et al., 2015).

Brucine could inhibit the growth of transplanted human gastric cancer cell line SGC-7901 and improve the weight loss of transplanted tumor in nude mice (Zhao et al., 2012). Agrawal et al., (2011) injected EAC cells into mice peritoneum to form ascites tumors, then treated with brucine at different doses. They found that brucine could induce anti-ascites tumor activity in a time-dose-dependent manner by reducing intraperitoneal angiogenesis and micro-vessel density *in vivo*.

### Anti-Inflammatory and Analgesic Effects

Within traditional Chinses medicine (TCM), the applications of *Nux-vomica* are closely related to its anti-inflammatory and analgesic effects, definitely for brucine. Hu et al., (2008) first used the improved Franz diffusion cell method to investigate the transdermal permeability of brucine solution at different concentrations *in vitro*, through observing the writhing number of mice within 25 min. The results showed that the middle and high dosage groups had obvious analgesic effect, and brucine could be quickly eliminated after percutaneous absorption. But because of its short half-life, brucine was not conducive to the lasting exertion of analgesic effect.

Meanwhile, brucine significantly inhibited the response induced by nociceptive heat and mechanical stimulation In acute pain models (Yu et al., 2019). In the carrageenan-induced rat paw swelling experiment, Yin et al., (2003) found that the amount of PGE2 in the foot swelling site of mice in the brucine 30 mg/kg experimental group was significantly lower than that in the blank control group (P < 0.05). Using paw retraction threshold (PWT) and paw retraction latency (PWL) to measure pain behavior in rats showed that brucine regulated peripheral analgesia through potassium channel (Li and Ren, 2019).

Brucine usually leads to gastrointestinal irritation and systemic toxicity by oral administration. A new type of gel permeable material system used to transport brucine could inhibit rheumatoid arthritis and significantly reduce the ear swelling caused by xylene in mice. It could also relieve the pain of formalin injection in formalin test indicating it had analgesic effect (Wu et al., 2017). Brucine could inhibit the proliferation of HFLS-RA cells by activating JNK signaling pathway. High dose brucine (> 0.5 mg/ml) could significantly reverse the proliferation induced by tumor necrosis factor α (TNF-α), and further inhibit the cell viability. In conclusion, brucine significantly inhibited the proliferation of HFLS-RA induced by TNF-α by activating JNK signaling pathway (Tang et al., 2019).

### Effects on Cardiovascular System

Li et al. (1997) observed the effects of brucine on action potential and contractile force of guinea pig papillary muscle by synchronous recording of contractile force with conventional microelectrode technique, displaying that brucine had an important effect on action potential induced by high K^+^ depolarized isoprenaline and histamine in isolated guinea pig papillary muscle. The mechanism might be that brucine blocked the Ca^2+^ channel in the myocardium then reduced the Ca^2+^ content in the myocardium, exhibiting a negative inotropic effect, and reducing the oxygen consumption of the myocardium. The above experiments showed that brucine had some antagonistic effect on arrhythmia.

### Other Pharmacological Activities

Brucine has paralysis effect on sensory nerve endings. When given a large dose, brucine can block nerve and muscle conduction, showing an arrow-like effect. It was showed that the expression of Bcl-2 and caspase-3 increased in the Wistar rats’ brain cells, so that Bcl-2 and caspase-3 were involved in the pathophysiological process of rat brucine poisoning (Lei et al., 2010).

It was investigated that brucine inhibited VEGF-induced cell proliferation, chemotactic motility, and the formation of capillary-like structures in HUVECs in a dose-dependent manner. The mechanism might be that brucine suppressed VEGF- induced p-VEGFR2 kinase activity, and down-regulated levels of VEGF, NO, IL-6, IL-8, TNF-α, and IFN-γ in HUVECs (Saraswati and Agrawal, 2013).

### Toxicity

Although *Nux-vomica* is widely used as a folk medicine, the poisonous properties have been known for centuries which limits its clinical application (Guo et al., 2018). As one of the ingredients of *Nux-vomica*, brucine is not only a medicinal component but also a toxic component (Wu et al., 2016b). Brucine has toxic effects on nervous system, immune system, urinary system, and digestive system. Serious central nervous system toxicity is the main obstacle of brucine clinical application. Toxicity has been shown to be related to dose and route of administration. In the toxic dose, brucine can cause severe convulsions, significantly increased blood pressure and even fatal poisoning. The route of administration also had a significant effect on the toxicity of brucine. In addition, it was found that the cytotoxicity of brucine was related to time and concentration, and brucine could be inserted into DNA double helix structure to interfere with DNA sequence (Liu et al., 2015).

## Prospects

In summary, brucine is the significant component of *Nux-vomica*, which has many pharmacological effects, particularly its anti-tumor, anti-inflammatory and analgesic effects have been studied most deeply. At present, most antitumor tests had been done *in vitro*, which is easier to control and operate. However, to accurately evaluate the antitumor effects, more experimentations *in vivo* need to be carried out.

Although brucine has an impressive preclinical profile in pharmacology research, serious toxicity limits its clinical applications. We suppose the novel pharmaceutic formulations, including target drug delivery systems (DDS), may help to solve the toxicity problems and enhance its efficacy. At present, there are some new formulations of brucine have been reported, such as transdermal preparations, including nanoparticles, liposomes, and so on. Brucine immuno-nanoparticles (BIN) and brucine-load solid nanoparticles both could successfully reduce adverse drug reactions and delay the release in the body (Qin et al., 2012a; Qin et al., 2012b; Deng et al., 2018). In addition, when treated with BIN, the proliferation, adhesion, invasion, and metastasis of SMMC-7721 cells were inhibited. BIN was a kind of polyethylene glycol-polyacic acid copolymer with anti-AFP monoclonal antibody prepared by chemical coupling technology (Qin et al., 2012b). And in nude mice model with hepatocellular carcinoma, the brucine immuno-nanoparticles could inhibit the growth of tumorsby significantly reducing the secretion of α-fetoprotein (AFP) (Qin et al., 2018). Brucine-load solid lipid nanoparticles (B-SLN) not only had the ability to improve the activity but also reduce the toxicity (Guan et al., 2017).

However, they are still mainly in pre-clinical studies. How to prepare more safe and effective new dosage forms to expand the clinical application will be the hot spot of brucine research in the future. Meanwhile, the pharmacological and toxicological mechanisms of brucine are still not very clear. It’s necessary to investigate the mechanism of brucine and establish an effective evaluation system in the future.

## Author Contributions

LL, RH, YW, and J-MJ wrote this manuscript. H-ZC and L-JZ revised the manuscript. XL presented the writing ideas and revised the manuscript.

## Funding

This work was supported by funds from the National Natural Science Foundation of China (No. 81903654, 81703755 and 81773941), Shanghai “Chenguang Program” of Education Commission of Shanghai Municipality (No.18CG46), Shanghai “Super Postdoctoral Fellow” incentive program (No. 2019334). “Yangfan Program” (No.19YF1449400) of Science and Technology Commission of Shanghai Municipality, Program for Professor of Special Appointment (Young Eastern Scholar) at Shanghai Institutions of Higher Learning, National Key Subject of Drug Innovation (2019ZX09201005-007) and National key R & D program for key research project of modernization of traditional Chinese medicine (2019YFC1711602).

## Conflict of Interest

The authors declare that the research was conducted in the absence of any commercial or financial relationships that could be construed as a potential conflict of interest.
